# Diagnostic Challenges in Visceral Leishmaniasis in a 17‐Month‐Old Female: A Case Report

**DOI:** 10.1002/ccr3.71447

**Published:** 2025-11-16

**Authors:** Ahmed Sheikh Sobeh, Manal Soufan, Tasneem Sarakbi, Alaa Jaddouh, Feras Alharroush

**Affiliations:** ^1^ Faculty of Medicine Hama University Hama Syria; ^2^ Department of Pediatric Hama Maternity and Children's Hospital Hama Syria

**Keywords:** amphotericin B, black fever, bone marrow aspiration, case report, kala‐azar, visceral leishmaniasis

## Abstract

Visceral leishmaniasis is an uncommon infectious disease. In this report, we present a case of VL in a 17‐month‐old child who presented with a fever and splenomegaly. After the patient failed to respond to several courses of antibiotics, a bone marrow aspirate confirmed the definitive diagnosis of VL, which was successfully treated with meglumine antimoniate. We emphasize the need to consider VL in any patient with persistent fever and splenomegaly, even if they come from a non‐endemic area.

## Graphical abstract

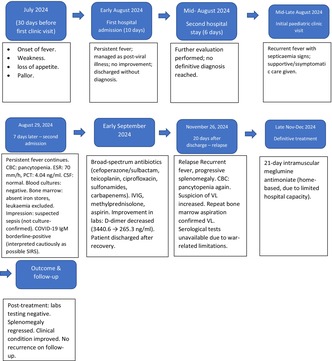

**Abbreviations**
CBC: complete blood count
CL: cutaneous leishmaniasis
CSF: cerebrospinal fluid
CT: computed tomography
DAT: direct agglutination test
ESR: erythrocyte sedimentation rate
IVIG: intravenous immunoglobulin
MAS: macrophage activation syndrome
ML: mucocutaneous leishmaniasis
PCR: polymerase chain reaction
PCT: procalcitonin test
SIRS: systemic inflammatory response syndrome
VL: visceral leishmaniasis

**Summary**

- Visceral leishmaniasis should be considered in any child with persistent fever and splenomegaly.
- Inconclusive tests warrant bone marrow aspiration, especially in resource‐limited contexts.
- Prompt diagnosis and therapy are critical to avoid severe complications and achieve favorable outcomes.

## 1 Introduction

Leishmaniasis is a diverse group of infectious diseases caused by Leishmania parasites [1, 2], which are intracellular protozoan parasites transmitted by female phlebotomine sand flies [3, 4, 5]. Leishmaniasis is endemic in the Middle East and Mediterranean countries, although more than 90% of cases occur in Bangladesh, India, Nepal, and other countries [6]. It has various clinical forms (mainly cutaneous, mucosal, or visceral), the most severe form being visceral leishmaniasis (VL) [6]. VL is also known as Kala‐azar or Black fever. It is a tropical and subtropical parasitic disease, also prevalent in poor communities where care is lacking [5]. VL is reported in over 70 countries, and it has become an endemic disease in more than 60 countries, but the incidence rate has decreased significantly over the past decade, from between 200,000 and 400,000 new cases in 2012 to between 50,000 and 90,000 in 2017 [4]. There are two species of Leishmania that cause VL: *L. donovani* in the Old World, including Asia, Africa, and Europe, and L. infantum (L. chagasi) in Latin America, Iran, Pakistan, and the Mediterranean countries (Syria) [2, 5, 6, 7]. The infection may be asymptomatic or an oligosymptomatic illness that either resolves spontaneously or evolves into active kala‐azar [3]. Common clinical symptoms of the disease include prolonged fever, weakness, loss of appetite, and hepatosplenomegaly [4]. Laboratory results may show pancytopenia and hyperglobulinemia [3, 4]. The gold standard for the diagnosis of VL is bone marrow aspiration, lymph node aspiration, or spleen biopsy [4]. All patients with VL should receive therapy with anti‐leishmanial drugs, including pentavalent antimony compounds or amphotericin B [4]. The importance of our clinical case lies in the similarity of the disease to other diseases, the difficulty of diagnosing it, its tendency to be overlooked, and the fact that early diagnosis and treatment are the two main measures to control leishmaniasis.

## 2 History of Presentation

**FIGURE 1:**
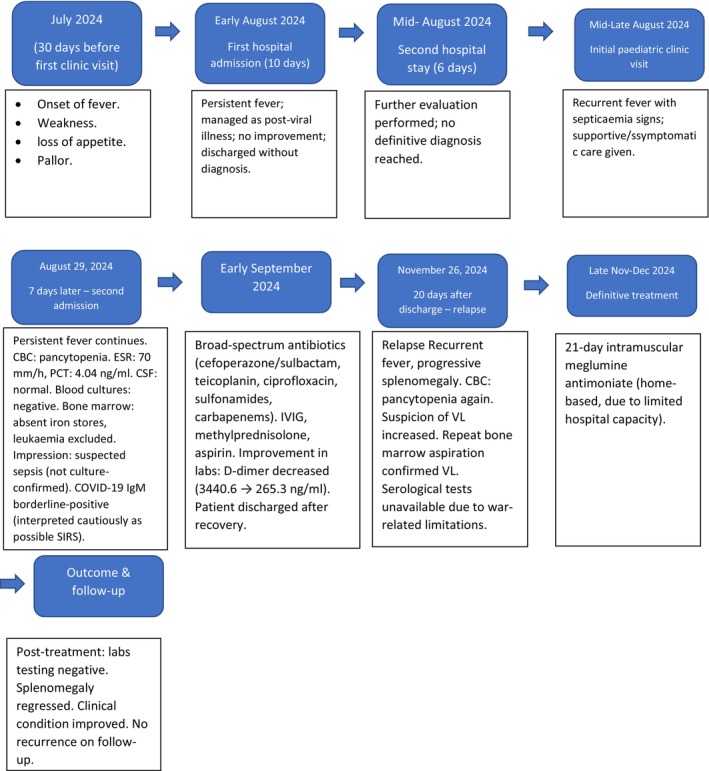
Timeline of a 17‐month‐old girl in whom visceral leishmaniasis was diagnosed after initial diagnostic challenges, finally confirmed by repeat bone marrow aspiration on November 26, 2024, and successfully treated at home.

We report the case of a 17‐month‐old Syrian female patient who was admitted to the hospital for 10 days because of fever, weakness, loss of appetite, and pallor. She was then referred to another hospital for 6 days for further evaluation. Investigations suggested a diagnosis of persistent fever after viral infection. The onset of these symptoms occurred 30 days before her initial visit to our pediatric clinic. She presented to our clinic with recurrent fever and signs of septicemia for which she received symptomatic treatment. The patient had no relevant medical or family history. However, 7 days later, she was admitted to the hospital because of recurrent fever (Figure 1, case timeline).

## 3 Differential Diagnosis

Identifying the underlying cause of unexplained fever can pose a considerable challenge. Typically, potential causes encompass a broad range of infectious and non‐infectious diseases, such as typhoid fever, malaria, brucellosis, miliary tuberculosis, leukemia, lymphoma, macrophage activation syndrome (MAS), and VL. In our case, VL was suspected, prompting a differential diagnosis among the aforementioned conditions.

## 4 Investigations

The complete blood count (CBC) revealed pancytopenia. Erythrocyte sedimentation rate (ESR) and procalcitonin (PCT) showed elevated values (ESR = 70 mm/h, PCT = 4.04 ng/mL). The cerebrospinal fluid (CSF) aspiration test result was normal, and the automated blood culture for aerobic and anaerobic bacteria was negative after 7 days of incubation. The remaining laboratory data of the patient are presented in Table 1. Hematological consultation was required when bone marrow aspiration showed the absence of iron stores on Prussian blue staining, and acute and chronic leukemia were excluded. In the clinical context of persistent fever and pancytopenia, sepsis was suspected; however, blood cultures remained negative. A few days later, the immunological COVID‐19 IgM test showed a borderline‐positive result (1.1 index; reference range: negative ≤ 0.9, borderline > 0.9 to < 1.1, positive ≥ 1.1). This finding was interpreted cautiously, suggesting a possible systemic inflammatory response syndrome (SIRS) in the context of COVID‐19, although its clinical significance remained uncertain.

**TABLE 1:** This table represents the values of the complete blood count test during the treatment from VL, where we can notice the improvement in the values of (RBCs, WBCs, PLT…).

Whole blood							
Para	Result					Unit	Reference range
	23–11	24–11	27–11	29–11	7–12		
WBC	2.6	4.48	2.98	3.47	6.50	10^3^/μL	4.00–10.00
RBC	2.61	3.41	3.10	3.55	3.89	10^6^/μL	3.50–5.50
HGB	6.1	9.2	8.4	9.3	9.6	g/dL	11.0–16.0
HCT	19.2	27.0	23.6	27.9	27.78	%	37.0–54.0
PLT	60	62	38	28	64	10^3^/μL	100–300
PCT	0.055	0.059	0.036	0.022	0.08	%	0.108–0.282

Abbreviations: HCT, hematocrit; HGB, hemoglobin; PCT, procalcitonin; PLT, platelet count; RBC, red blood cell count; WBC, white blood cells.

## 5 Management

Antibiotic therapy with cefoperazone + sulbactam, teicoplanin, ciprofloxacin, sulfonamides, and carbapenems was initiated for the patient due to clinical findings indicating sepsis.

Moreover, the patient was prescribed intravenous immunoglobulin (IVIG), alongside methylprednisolone and aspirin administered separately, after which the laboratory test showed improvement in our patient's case (D‐Dimer value decreased from 3440.6 ng/mL before treatment to 265.3 ng/mL after treatment; reference range up to 500 ng/mL). After improvement in the general condition and laboratory test results, the patient was discharged from the hospital. After 20 days, our patient developed a recurrent fever with progressive splenomegaly. CBC revealed pancytopenia again. After excluding most differential diagnoses, suspicion of VL was raised, and a second bone marrow aspiration was performed on November 26, 2024, which confirmed the diagnosis of VL. Unfortunately, serological tests for VL (such as rK39, DAT, or ELISA) were not available at the time due to the war‐related resource limitations in Syria. Therefore, bone marrow aspiration served as the only definitive diagnostic method. A 21‐day course of intramuscular meglumine antimoniate at a dose of 20 mg/kg per day was administered at home under medical supervision due to limited hospital resources during the war.

## 6 Outcome and Follow‐Up

After completing the treatment, the tests were repeated and were negative. No recurrence was reported, with improvement in her general condition and regression of the splenomegaly (Tables 1 and 2).

**TABLE 2:** This table shows the improvement in the values of C‐reactive protein in addition to liver function tests, including ALT and AST.

Chemistry	Result	Unit	Reference range
23‐11‐2024			
CRP	164.58	mg/dL	< 5
27‐11‐2024			
CRP	98	mg/dL	< 5
8‐12‐2024			
CRP	54	mg/dL	< 5

Liver function test			
26‐11‐2024			
ALT	11	U/L	0–40
AST	37	U/L	0–37

Abbreviations: ALT, alanine transaminase; AST, aspartate aminotransferase; CRP, C‐reactive protein.

## 7 Discussion

Leishmaniasis is a common vector‐borne parasitic disease caused by intracellular protozoa of the Trypanosomatida genus Leishmania [2, 4, 7]. Leishmaniasis is a serious public health issue that threatens human health [2]. There are three clinical forms of leishmaniasis: cutaneous (CL), mucocutaneous (ML), and VL, which is the most serious form due to its spread through the reticuloendothelial system [2, 6]. After the parasite enters the skin, it spreads to the liver and spleen via the blood [6, 8]. The clinical manifestations of VL vary depending on the patient's immunity [6]. Patients may be asymptomatic (subclinical), with mild fever, weakness, weight loss, and hepatosplenomegaly [9]. In late‐stage kala‐azar, the patient presents with high‐grade fever above 40°C, gross wasting, visceral hypertrophy, jaundice, edema, ascites, severe pancytopenia, and a positive delayed‐type hypersensitivity test (Montenegro test) [3, 4, 8, 9, 10, 11]. Severe anemia may lead to heart failure. Bleeding episodes, especially epistaxis, are frequent [3]. Secondary bacterial infections aggravate the disease, which is frequently a cause of death [3]. In our case, the child had a fever that was unresponsive to antipyretics, with fatigue. Clinical examination revealed a high fever, weakness, pallor, and splenomegaly. The nonspecific nature of these symptoms, which overlapped with those of other diseases, posed difficulties in preliminary diagnosis. To diagnose leishmaniasis, clinical signs must be found in addition to serological tests [10]. Any hepatosplenomegaly can be detected by clinical examination or using ultrasound or computed tomography (CT) [6]. Laboratory tests, such as a complete blood count (CBC), reveal anemia (hemoglobin, 5–8 g/dL), thrombocytopenia, leukopenia (2000–3000 cells/μL), and hyperglobulinemia, although these tests are non‐specific [6]. In general, noninvasive procedures such as polymerase chain reaction (PCR) are recommended first, as they have a high sensitivity (80–100%) [6, 12]. Serologic testing by enzyme immunoassay, indirect fluorescence assay, or direct agglutination test (DAT) is very useful. Immunochromatographic strip tests using a recombinant antigen (K39) have high diagnostic sensitivity and specificity [3, 4]. However, at the onset of infection, diagnosing VL remains challenging, particularly because the parasite is confined to the liver, spleen, and bone marrow. Therefore, invasive methods such as bone marrow aspiration, lymph node aspiration, or spleen biopsy are required, with a high sensitivity rate (93–99%) [5, 6, 10, 12]. In our patient, CBC showed pancytopenia, but cerebrospinal fluid (CSF) puncture was negative. Ultrasonography revealed an enlarged spleen by about 1 cm from normal size. Bone marrow aspiration from the iliac bone was also performed, and a significant number of Leishmania parasites were observed within the macrophages as well as extracellularly, confirming the diagnosis. For management, liposomal amphotericin B is the gold standard for treating VL, although antimonial compounds have been used for decades as a treatment for VL [4, 7, 12], and remain an alternative treatment for patients who cannot tolerate other treatments [6]. In Syria, meglumine antimoniate (Glucantime) is the main treatment, especially in pediatric patients, as it is considered effective, safe, and inexpensive [5, 13]. In our case, because the initial diagnosis was difficult and bacterial infection was suspected, the patient was treated with empiric intravenous antibiotic therapy. However, after the diagnosis of VL, she was treated with intramuscular meglumine antimoniate at a dose of 20 mg/kg per day for 21 days. Despite the rarity of VL and the difficulty in suspecting it owing to its non‐specific symptoms, it should be considered as a differential diagnosis for such symptoms, and appropriate investigations should be performed. This case was managed in a resource‐limited setting during the conflict period in Syria, before relative stability was restored. Standard serological tests for VL (rK39, DAT, ELISA) and confirmatory molecular testing for COVID‐19 were not available, and the diagnosis relied on bone marrow aspiration. The interpretation of sepsis was made clinically in the absence of advanced microbiological facilities. In addition, due to limited hospital capacity, anti‐leishmanial therapy was continued at home rather than under inpatient monitoring.

## 8 Conclusion

There are three clinical forms of leishmaniasis: cutaneous (CL), mucocutaneous (ML), and visceral (VL). This is a serious public health issue that threatens human health. VL is the most serious form of leishmaniasis and should be included in the differential diagnosis of any child with fever, splenomegaly, or anemia. The correct diagnosis depends on the exclusion of other differential diagnoses using laboratory and immunological tests, in addition to confirmation by visualization of the Leishmania parasite during bone marrow aspiration. Choosing appropriate treatment tailored to each patient in their specific context leads to better management and outcomes.

## Author Contributions

**Ahmed Sheikh Sobeh:** data curation, writing – original draft, writing – review and editing. **Manal Soufan:** conceptualization, data curation, project administration, writing – original draft, writing – review and editing. **Tasneem Sarakbi:** data curation, writing – original draft, writing – review and editing. **Alaa Jaddouh:** data curation, writing – original draft, writing – review and editing. **Feras Alharroush:** investigation, supervision, writing – review and editing.

## Consent

Written informed consent was obtained from all the patients for publication and accompanying images. A copy of the written consent form is available for review by the Editor‐in‐Chief of the journal upon request.

## Conflicts of Interest

The authors declare no conflicts of interest.

## Data Availability

The materials used in this study are available from the corresponding authors upon request.
